# Tetrahydrocannabinol Induces Brain Mitochondrial Respiratory Chain Dysfunction and Increases Oxidative Stress: A Potential Mechanism Involved in Cannabis-Related Stroke

**DOI:** 10.1155/2015/323706

**Published:** 2015-01-14

**Authors:** Valérie Wolff, Anna-Isabel Schlagowski, Olivier Rouyer, Anne-Laure Charles, François Singh, Cyril Auger, Valérie Schini-Kerth, Christian Marescaux, Jean-Sébastien Raul, Joffrey Zoll, Bernard Geny

**Affiliations:** ^1^EA 3072, Fédération de Médecine Translationnelle de Strasbourg, Institut de Physiologie, Université de Strasbourg, 67000 Strasbourg, France; ^2^Unité Neuro-Vasculaire, Service de Neurologie, Hôpitaux Universitaires de Strasbourg, 67000 Strasbourg, France; ^3^CNRS, ICube UMR-7357, Fédération de Médecine Translationnelle de Strasbourg, Université de Strasbourg, 67000 Strasbourg, France; ^4^Service de Physiologie et d'Explorations Fonctionnelles, Pôle de Pathologie Thoracique Hôpitaux Universitaires, NHC, CHRU de Strasbourg, 67000 Strasbourg Cedex, France; ^5^UMR CNRS 7213, Laboratoire de Biophotonique et Pharmacologie, Faculté de Pharmacie, Université de Strasbourg, 67401 Illkirch, France; ^6^Laboratoire de Toxicologie, Institut de Médecine Légale, Université de Strasbourg, 67000 Strasbourg, France

## Abstract

Cannabis has potential therapeutic use but tetrahydrocannabinol (THC), its main psychoactive component, appears as a risk factor for ischemic stroke in young adults. We therefore evaluate the effects of THC on brain mitochondrial function and oxidative stress, key factors involved in stroke. Maximal oxidative capacities *V*_max_ (complexes I, III, and IV activities), *V*_succ_ (complexes II, III, and IV activities), *V*_tmpd_ (complex IV activity), together with mitochondrial coupling (*V*_max_/*V*_0_), were determined in control conditions and after exposure to THC in isolated mitochondria extracted from rat brain, using differential centrifugations. Oxidative stress was also assessed through hydrogen peroxide (H_2_O_2_) production, measured with Amplex Red. THC significantly decreased *V*_max_ (−71%; *P* < 0.0001), *V*_succ_ (−65%; *P* < 0.0001), and *V*_tmpd_ (−3.5%; *P* < 0.001). Mitochondrial coupling (*V*_max_/*V*_0_) was also significantly decreased after THC exposure (1.8±0.2 versus 6.3±0.7; *P* < 0.001). Furthermore, THC significantly enhanced H_2_O_2_ production by cerebral mitochondria (+171%; *P* < 0.05) and mitochondrial free radical leak was increased from 0.01±0.01 to 0.10±0.01% (*P* < 0.001). Thus, THC increases oxidative stress and induces cerebral mitochondrial dysfunction. This mechanism may be involved in young cannabis users who develop ischemic stroke since THC might increase patient's vulnerability to stroke.

## 1. Introduction

Cannabis is now largely considered for its therapeutic potential and is the most widely used recreational drug in the world [1–4]. The principal psychoactive cannabinoid in cannabis is tetrahydrocannabinol (THC), and although cannabis is considered by many people as having few negative side-effects, severe cardiovascular complications were described such as myocardial infarction, sudden death, peripheral arteritis, and stroke [5–7]. Concerning stroke, we described in a prospective series that there was a link between ischemic stroke (IS) and cannabis use in young patients [8].

However, little is known regarding the potential mechanisms implied in cannabis-related stroke. Besides vascular alterations, cannabis might directly affect brain mitochondrial function and oxidative stress, both factors previously shown to be involved in stroke [9–11]. Indeed, mitochondria are a main source of adenosine triphosphate (ATP) production and are particularly involved in the balance between cell survival and cell death. Most cellular energy is obtained through oxidative phosphorylation, a process requiring the action of a set of respiratory enzyme complexes located in the inner mitochondrial membrane. It appears pertinent to study the activities of the different mitochondrial respiratory chain complexes, since mitochondrial respiratory chain complexes activities impairment is observed in many acute and chronic diseases [12–17]. This study is also justified because THC has been previously shown to decrease brain energetic metabolism [18].

Further and interestingly, increased reactive oxygen species (ROS) production leading to oxidative stress is involved in acute IS [9–11, 19, 20]. Oxidative stress not only is a major cause of endothelium dysfunction in the cerebral circulation [21] but can also directly affect mitochondrial function. Thus, as a vital organ rich in mitochondria and with high oxygen use, the brain is prone to oxidative stress and might be particularly sensitive to mitochondrial damage [22].

To challenge the hypothesis that THC might participate to cannabis-related stroke, we first determined the effects of THC on maximal brain mitochondrial respiration rate, and then analyzed precisely complexes I, II, III, and/or IV activities of the mitochondrial respiratory chain together with mitochondrial coupling. We also determined whether THC might increase ROS production in brain mitochondria and measured the mitochondrial free radical leak.

## 2. Material and Methods

### 2.1. Material, Reagents and Animals

Synthetic THC (25 mg/mL in ethanol), mitochondrial complexes substrates and/or inhibitors such as glutamate, malate, amytal, ADP, succinate, ascorbate, antimycine, and N,N,N′,N′-tetramethyl-p-phenylenediaminedihydrochloride (tmpd) were acquired from Sigma-Aldrich, France. THC was successively diluted in ethanol as needed and Amplex Red and horseradish peroxidase (HRP) were acquired by Invitrogen. All other chemicals used were of the highest grade commercially available.

Ten male Wistar rats (weight 438 to 500 grams; age 13 weeks) were housed in a neutral temperature environment (22°  ± 2°C), on a 12 : 12 hours photoperiod, and were provided food and water* ad libitum*. This investigation was carried out in accordance with the Helsinki accords for human treatment of animals during experimentation. Rats were submitted to general anesthesia with 3% isoflurane and oxygen (2 L/min) in an induction chamber (Minerve, Esternay, France) and were then decapitated. Brains were excised and cleaned and then immediately used for the study of respiratory parameters.

### 2.2. Extraction of Brain Mitochondria

Extraction of brain mitochondria was performed as previously reported [23, 24]. All experimental steps were carried on ice. A piece of brain was placed into buffer A containing 50 mM tris, 1 mM ethylene glycol tetraacetic acid (EGTA), 70 mM sucrose, 210 mM mannitol, pH 7.40 at +4°C. Tissues were finely minced with scissors, placed in buffer A and homogenized with a gentleMACS Dissociator (Miltenyi Biotec, Bergisch Gladbach, Germany). Then, the homogenate was centrifuged at 1300 ×g for 3 minutes, 4°C. The supernatant was centrifuged at 10000 ×g for 10 minutes, 4°C, to sediment mitochondria. Finally, the mitochondrial pellet was washed twice and then suspended in 50 mM Tris, 70 mM sucrose, and 210 mM mannitol, pH 7.4 at +4°C. Protein content was routinely assayed with a Bradford assay using bovine serum albumin (BSA) as a standard [24].

### 2.3. Dose-Effect Curve of THC on Brain Mitochondria Maximal Oxidative Capacity

Before measurement, 3 mL of solution M containing 100 mM KCl, 50 mM Mops, 1 mM EGTA, 5 mMKpi, and 1 mg/mL defatted BSA was added to the oxygraph chambers for 10 minutes. Then, isolated mitochondria of brain (0.30 mg) were placed in the oxygraph chambers (using Clark oxygen electrodes) with glutamate (5 mM) and malate (5 mM) as substrates. The temperature was maintained at +25°C.

Adenosine diphosphate (ADP, 2 mM) was then added to measure maximal oxidative capacity (*V*_max⁡_). After 1.5 minutes, increasing concentrations of THC were added to the respiration solution. The ranges of THC concentrations were 10^−5^, 2∗10^−5^, 3∗10^−5^, 4∗10^−5^, 5∗10^−5^, and 6∗10^−5^ M. The objective was to determine the dilution of THC in ethanol needed to inhibit at least 50% of the maximal mitochondrial respiration (*V*_max⁡_) and the half maximal inhibitory concentration (IC_50_).

### 2.4. Mitochondrial Coupling Determination

After the determination of the basal oxygen consumption (*V*_0_), the maximal brain mitochondrial respiration rates were measured in the presence of ADP as a phosphate acceptor (*V*_max⁡_). The degree of coupling between oxidation and phosphorylation was inferred from the acceptor control ratio *V*_max⁡_/*V*_0_ (ACR).

### 2.5. Mitochondrial Respiratory Chain Complexes Activities

When *V*_max⁡_ was recorded, electron flow went through complexes I, III, and IV [25]. Complex I was blocked with amytal (0.02 mM) and complex II was stimulated with succinate (25 mM). Mitochondrial respiration in these conditions allowed determining complexes II, III, and IV activities (*V*_succ_).

After that, complex III was blocked by antimycine (1.8 *μ*g/mL) and tmpd (0.5 mM) and ascorbate (0.5 mM) were added as artificial electron donors to cytochrome c. In these conditions, the activity of cytochrome c oxydase (complex IV) was determined as an isolated step of the respiratory chain (*V*_tmpd_).

In order to evaluate the effect of THC on the different mitochondrial respiratory chain complexes activities, THC was added 90 sec respectively after *V*_max⁡_, *V*_succ_, and *V*_tmpd_. We used the dilution of THC determined by the dose-effect curve which inhibited at least 50% of *V*_max⁡_. Forty-five independent analyses of mitochondrial respiration were performed. Each mean value was obtained from 15 independent measurements in triplo.

### 2.6. Measurements of the Production of H_2_O_2_ by Brain Mitochondria

Production of H_2_O_2_ with and without THC was measured with Amplex Red which reacted with H_2_O_2_ in a 1 : 1 stoichiometry catalyzed by HRP to yield the fluorescent compound resorufin and a molar equivalent of O_2_ [26, 27]. Resorufin has excitation and emission wavelengths of 563 nm and 587 nm, respectively, and is extremely stable once formed. Fluorescence was measured continuously with a Fluoromax 3 (Jobbin Yvon) spectrofluorometer with temperature control and magnetic stirring. After a baseline (reactants only) was established, the reaction was initiated by adding brain isolated mitochondria (0.30 mg) to 600 *μ*L of a buffer (KCl 125 mM, KH_2_PO_4_ 4 mM, NaCl 14 mM, HEPES-NaOH 20 mM, MGCl_2_ 1 mM, EGTA 0.020 mM, and 0.2% fatty acids free BSA, pH 7.2).

H_2_O_2_ production was first determined with glutamate (10 mM) and malate (2 mM), with ADP (2 mM) used as substrates. We added THC and compared H_2_O_2_ production without and with THC. The results were reported in pmol/min/mg protein. Values are the means ± SEM of 12 independent experiments.

### 2.7. Measurement of the Mitochondrial Free Radical Leak (FRL)

H_2_O_2_ production and O_2_ consumption were measured in parallel in the same sample under similar experimental conditions. This allowed the calculation of the fraction of electrons out of sequence which reduce O_2_ to ROS in the respiratory chain (the percent of free radical leak) instead of reaching cytochrome oxidase to reduce O_2_ to water [12, 26, 27]. Because 2 electrons are needed to reduce 1 mole of O_2_ to H_2_O_2_, whereas 4 electrons are transferred in the reduction of 1 mole of O_2_ to water, the percent free radical leak was calculated as the rate of H_2_O_2_ production divided by two times the rate of O_2_ consumption, and the result was multiplied by 100. The FRL was calculated before and after THC exposure.

### 2.8. Statistical Analysis

Results are expressed as mean ± SEM. Statistical analyses were performed using Student's *t*-test, one-way repeated measures, or two-way ANOVA followed by Tukey's posttest (GraphPad Prism 5, Graph Pad Software, Inc., San Diego, CA, USA). Statistical significance was displayed as ^*^ *P* < 0.05 or ^**^ *P* < 0.01 or ^***^ *P* < 0.001.

## 3. Results

### 3.1. Dose-Dependent Inhibition of Maximal Brain Mitochondrial Respiration by THC

**Figure 1:**
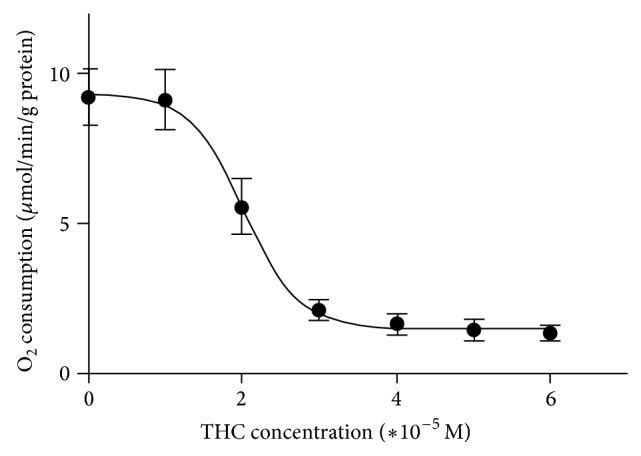
THC decreased brain mitochondrial maximal oxidative capacity: dose-response curve. Effects of ranges concentrations of THC (10^−5^ to 6∗10^−5^ M) on brain mitochondrial maximal oxygen consumption, measured using glutamate and malate as substrates. Values are expressed in *μ*mol/min/g protein.

Dose-dependent inhibition of respiration by THC was demonstrated in the maximal oxidative capacities (*V*_max⁡_) study and data were fit to a 4-parameter sigmoidal dose-response model to determine half-maximal inhibitory concentration (IC_50_) values. THC exhibited an apparent IC_50_ value of about 2∗10^−5^ M (Figure 1).

### 3.2. THC Impaired All Complexes of the Brain Mitochondrial Respiratory Chain and Decreases Mitochondrial Coupling

**Figure 2:**
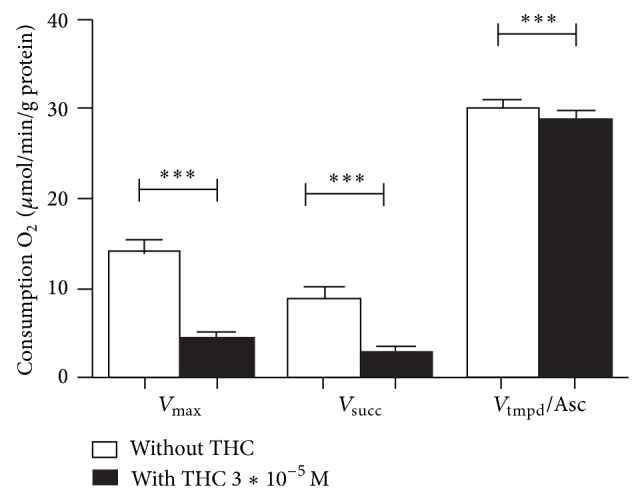
THC impaired complexes I, II, III, and IV activities of the brain mitochondrial respiratory chain. Effects of 3∗10^−5^ M of THC (black graphs) on brain mitochondrial respiratory chain complexes activities as compared to control values (white graphs). *V*_max⁡_ reflects complexes I, III, and IV activities and is measured using ADP. *V*_succ_ reflects complexes II, III, and IV activities and is measured using succinate. *V*_tmpd_ reflects complex IV activity and is measured using tmpd and ascorbate as mitochondrial substrates. THC: tetrahydrocannabinol. Data are means ± SEM and ^***^ *P* < 0.001.

Figure 2 represents brain mitochondrial respiratory chain complexes activities before and after 3∗10^−5^ M of THC exposure. Baseline activities are represented by *V*_max⁡_, *V*_succ_, and *V*_tmpd_ and mitochondrial coupling. The maximal oxidative capacities, *V*_max⁡_ (*n* = 15), reflecting complexes I, III, and IV activities was 13.9 ± 1.3 *μ*mol O_2_/min/g protein. *V*_succ_ (*n* = 15), reflecting complexes II, III, and IV activities, was 8.7 ± 1.4 *μ*mol O_2_/min/g protein. *V*_tmpd_ (*n* = 15), reflecting complex IV activity, was 29.9 ± 0.9 *μ*mol O_2_/min/g protein.

*V*_max⁡_ (*n* = 15), *V*_succ_ (*n* = 15), and *V*_tmpd_ (*n* = 15) were significantly decreased after exposure to THC 3∗10^−5^ M in brain isolated mitochondria (resp., 4.4 ± 0.7 *μ*mol O_2_/min/g protein versus 13.9 ± 1.3; *P* < 0.001; 2.8 ± 0.5 versus 8.7 ± 1.4 *μ*mol O_2_/min/g protein; *P* < 0.001; 28.8 ± 1 versus 29.9 ± 0.9 *μ*mol O_2_/min/g protein; *P* < 0.001 with and without exposure, resp.).

The effect of THC on the respiratory chain might be linked to an effect on complexes I, II, and III rather than on complex IV, because THC reduced *V*_max⁡_ by 71%, which reflects I, III, and IV activities, reduced *V*_succ_ by 68%, which reflects complexes II, III, and IV activities and reduced *V*_tmpd_ by 3.5% which reflects complex IV activity.

Finally, mitochondrial coupling (*V*_max⁡_/*V*_*o*_) was also significantly decreased after adjunction of THC (1.8 ± 0.2 versus 6.3 ± 0.7; *P* < 0.001).

### 3.3. THC Increased Brain H_2_O_2_ Production

**Figure 3:**
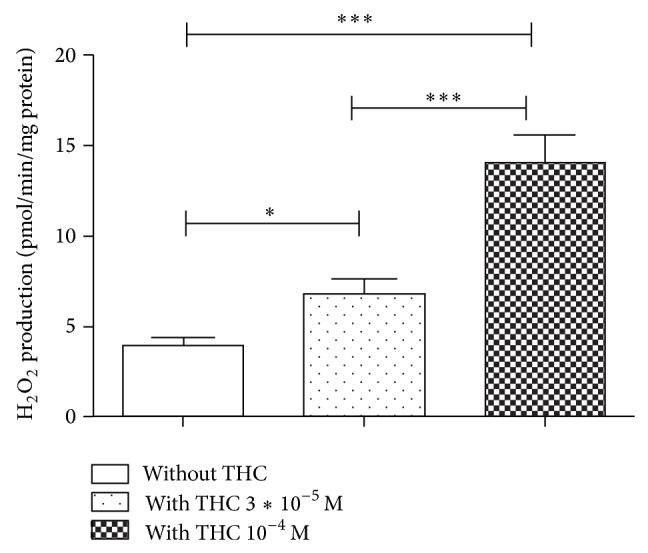
THC increased brain mitochondrial H_2_O_2_ production. Effects of 3∗10^−5^ M and 10^−4^ M of THC on brain mitochondria H_2_O_2_ production, as compared to control values (white graph). THC: tetrahydrocannabinol. Values are expressed in pmol/min/mg protein.

To assess if the decreased brain mitochondria respiration induced by THC was related to the generation of ROS, we determined H_2_O_2_ production by brain mitochondria without and with THC in a concentration of 3∗10^−5^ M or 10^−4^ M on 12 independent experiments. The addition of 3∗10^−5^ M of THC and 10^−4^ M of THC significantly increased H_2_O_2_ by, respectively, 171% and 371% in comparison with baseline production (6.7 ± 0.8 versus 3.9 ± 0.4 pmol/min/mg; *P* < 0.05 and 14.1 ± 1.4 versus 3.9 ± 0.4 pmol/min/mg; *P* < 0.001) (Figure 3).

### 3.4. THC Increased the Mitochondrial Free Radical Leak in the Brain

**Figure 4:**
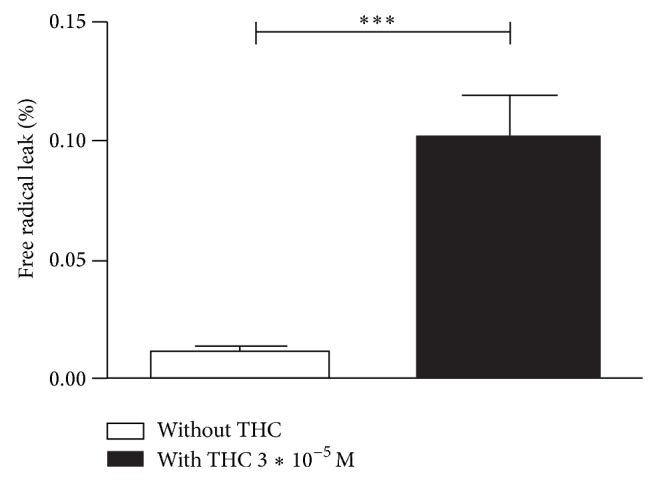
THC increased the free radical leak in brain mitochondria. Effect of 3∗10^−5^ M of THC on the free radical leak (FRL). FRL corresponds to the fraction of electrons out of sequence which reduces O_2_ to ROS in the respiratory chain (the percent of free radical leak) instead of reaching cytochrome oxidase. THC: tetrahydrocannabinol. Control FRL without THC (white graph) and FRL after THC exposure (black graph).

The mitochondrial free radical leak (FRL) was increased after THC addition from 0.01 ± 0.01 to 0.10 ± 0.01%, *P* < 0.001 (Figure 4).

## 4. Discussion

The main results of this study are to show that THC has a direct dose-dependent toxic effect on brain mitochondria and to demonstrate for the first time that THC mainly inhibits complexes I, II, and III of the mitochondrial respiratory chain and decreases mitochondrial coupling. Furthermore, THC increases ROS production by the brain, which likely participates in its toxicity.

Cannabis is the most frequent illicit substance used in the world and has been associated with cardiovascular complications, especially stroke in young adults [6, 7]. Vascular effects might be involved and cerebral arterial stenoses have been observed but this could not be the sole mechanism and the precise actions of cannabis on brain in patients who develop a stroke are not determined. Particularly, THC, which is the main component of cannabis, has been shown to decrease mitochondrial oxygen consumption in oral cancer cells [28] and in human sperm [29]. In only few experimental reports, THC has been described to induce mitochondrial dysfunction* in vitro*, reducing oxygen consumption on several organs including the heart [30, 31], the liver [32], the skeletal muscle [33], or the brain [31, 33].

Recently, synthetic cannabinoids (known as K2) has been shown to result in ischemic stroke [34], supporting the need to further evaluate the potential toxicity of synthetic THC on brain mitochondria. In the present study, isolated mitochondria were incubated with different concentrations of THC and we demonstrated that THC globally impaired mitochondrial respiratory chain complexes activities. This is in accordance with the literature, similar results being observed* in vitro* on the brain of mice and rat [31, 33].

To go further, we analyzed the specific cellular effect of THC on different complexes of the mitochondrial respiratory chain. We showed for the first time that THC significantly reduced *V*_max⁡_ (71% inhibition) reflecting complexes I, III, and IV activities, *V*_succ_ (68% inhibition) reflecting II, III, and IV activities, and more slightly *V*_tmpd_ reflecting complex IV activity. Thus, THC has a main deleterious effect on complexes I, II, and III of the mitochondrial respiratory chain. This is globally consistent with the data of Athanasiou et al. showing on heart mitochondria in rat that THC may affect complexes I, II, and III depending on the concentration of THC used [30].

Additionally, we observed that mitochondrial coupling was decreased after THC adjunction, further supporting its toxic effects on the brain.

To get further knowledge on the mechanism potentially involved in the deleterious effect of THC on brain mitochondria, we determined ROS production as inferred by hydrogen peroxide change. Interestingly, THC induced a significant production of ROS (+171%). Since mitochondria are both causes and targets of ROS, mitochondrial production of H_2_O_2_ might be increased as a result of cannabis-related mitochondrial dysfunction.

Accordingly, the free radical leak increased after THC exposure, supporting that the fraction of electrons which reduce O_2_ to ROS in the respiratory chain [35] were greater in presence of cannabis. Thus, mitochondria likely participated in the ROS overproduction seen in the presence of THC.

This appears important since oxidative stress is a pathophysiological mechanism involved in stroke and since the brain is particularly vulnerable to oxidative stress with few protective antioxidant mechanisms [22]. From another point of view, Bartova and Birmingham reported that THC reduces NADPH activity, suggesting a decreased ROS production [31]. However the author did not measure directly ROS synthesis in the mitochondria. In our study we found both an altered function of the mitochondrial respiratory chain and an increased synthesis of H_2_O_2_ when using THC. Taken all data in consideration, one could speculate that THC might have different effects on various sources of ROS production and/or on ROS antioxidant defense and that the balance leads to an increased oxidative stress.

Our data are likely to be pertinent in the clinical setting since THC and its main metabolite 11-hydroxy-delta9-THC are highly lipophilic and cross the blood-brain barrier [36]. Thus, THC concentration has been observed in animals [36] and human brains [37, 38]. Accordingly, although the effects of THC on brain respiration is controversial after a single intraperitoneal dose [18, 39, 40], Costa and Colleoni reported a decrease in oxygen consumption when repeated doses of THC were administrated [18]. Such decrease in oxygen consumption in chronic treatment by THC indicated low ATP production, little disposable energy and consequent neuronal damage. In favor of this hypothesis, we observed in our previous prospective study that all patients who developed a stroke related to cannabis use were chronic abusers [8]. Further, inhaled marijuana smoke has been shown to disrupt mitochondrial energetics in pulmonary epithelial cells* in vivo* [41].

Taken together, these data suggest that the deleterious effect of THC on brain mitochondria could be linked to its ability to generate oxidative stress and that this mechanism may be involved in young cannabis users who develop a stroke.

This study presents some limitations. Although the* in vitro* approach gives a better control of the amount of THC that actually reaches the mitochondria itself, to approach real life conditions of cannabis consumption needs THC administration* in vivo*. Accordingly, intraperitoneal administration of THC allowed assessing attention functions or addiction in rats [42, 43]. Such route may nevertheless lead to controversial results when mitochondrial function is examined [18, 39, 40]. Thus, further studies aiming to closely resemble the effects of cannabis in the brain* in vivo* are needed, investigating not only intraperitoneal but also intravenous and/or inhalation routes often used by humans.

## 5. Conclusion

THC exposure alters brain maximal oxidative capacity. It impairs mainly the complexes I, II, and III of the mitochondrial respiratory chain and mitochondrial coupling. THC also increases brain ROS production and mitochondrial free radical leak.

Both mitochondrial dysfunction and oxidative stress are key events during stroke, suggesting that THC might increase patient's vulnerability to stroke and that further investigations would be helpful to determine whether mitochondrial protection and antioxidants might decrease THC-related neuronal damage in cannabis-induced stroke.
